# Mortality and years of life lost by colorectal cancer attributable to physical inactivity in Brazil (1990–2015): Findings from the Global Burden of Disease Study

**DOI:** 10.1371/journal.pone.0190943

**Published:** 2018-02-01

**Authors:** Diego Augusto Santos Silva, Mark Stephen Tremblay, Maria de Fatima Marinho de Souza, Meghan Mooney, Mohsen Naghavi, Deborah Carvalho Malta

**Affiliations:** 1 Research Center in Kinanthropometry and Human Performance, Federal University of Santa Catarina, Florianopolis, SC, Brazil; 2 Children’s Hospital of Eastern Ontario Research Institute, Ottawa, ON, Canada; 3 Department of Surveillance of Noncommunicable Diseases, and Injuries, and Health Promotion, Ministry of Health, Brasília, DF, Brazil; 4 Institute for Health Metrics and Evaluation, University of Washington, Seattle, WA, United Sates of America; 5 Department of Maternal and Child Nursing and Public Health, School of Nursing, Federal University of Minas Gerais, Belo Horizonte, MG, Brazil; Karolinska Institutet, SWEDEN

## Abstract

**Introduction:**

The aims of this study were to estimate all-cause and cause-specific mortality and years of life lost, investigated by disability-adjusted life-years (DALYs), due to colorectal cancer attributable to physical inactivity in Brazil and in the states; to analyze the temporal trend of these estimates over 25 years (1990–2015) compared with global estimates and according to the socioeconomic status of states of Brazil.

**Methods:**

Databases from the Global Burden of Disease Study (GBD) for Brazil, Brazilian states and global information were used. It was estimated the total number and the age-standardized rates of deaths and DALYs for colorectal cancer attributable to physical inactivity in the years 1990 and 2015. We used the Socioeconomic Development Index (SDI).

**Results:**

Physical inactivity was responsible for a substantial number of deaths (1990: 1,302; 2015: 119,351) and DALYs (1990: 31,121; 2015: 87,116) due to colorectal cancer in Brazil. From 1990 to 2015, the mortality and DALYs due to colorectal cancer attributable to physical inactivity increased in Brazil (0.6% and 0.6%, respectively) and decreased around the world (-0.8% and -1.1%, respectively). The Brazilian states with better socioeconomic indicators had higher rates of mortality and morbidity by colorectal cancer due to physical inactivity (p<0.01). Physical inactivity was responsible for deaths and DALYs due to colorectal cancer in Brazil.

**Conclusions:**

Over 25 years, the Brazilian population showed more worrisome results than around the world. Actions to combat physical inactivity and greater cancer screening and treatment are urgent in the Brazilian states.

## Introduction

Colon and rectum cancer (called colorectal cancer) is the third most lethal type of cancer in women and the fourth in men in Brazil [1]. In the United States, this type of cancer is the second most lethal [2]. This neoplasm has a multifactorial etiology that includes both genetic and modifiable lifestyle factors [3].

Among the lifestyle factors, physical inactivity stands out because it interacts with some genes that influence the onset of colorectal cancer, which potentiates the onset of the disease [3]. In addition, physical inactivity is a risk factor for obesity, which is another independent predictor of colorectal cancer [4,5]. Dose-response evidence has shown that regular physical activity can reduce the onset of colorectal cancer by 20–25% in both men and women.^5^ Mechanisms by which physical activity reduces the risk of colorectal cancer, were not entirely clear, albeit assumptions such as changes in the material in gastrointestinal transmit time, changes in immune function as well as changes in prostaglandin levels, insulin, insulin-like growth factors, bile acid secretion, serum cholesterol and pancreatic and gastrointestinal hormone profiles have been forwarded [4–6].

Although the benefits of physical activity for health are unequivocal, data from the Global Burden of Disease (GBD) study revealed that physical inactivity is the second greatest health risk factor to which the Brazilian population is exposed [7]. Complementing this information, the literature reinforces that physical inactivity is an independent risk factor for cancer morbidity and mortality and for non-communicable diseases more generally [8].

The aim of this study was to estimate the all-cause and cause-specific mortality and years of life lost, investigated by disability-adjusted life-years (DALYs), due to colorectal cancer attributable to physical inactivity in Brazil and in the states. A second aim was to analyze the temporal trend of these estimates over 25 years (1990–2015) compared with global estimates and according to the socioeconomic status of the states of Brazil.

## Material and methods

### Study overview

GBD 2015 includes an annual assessment covering 195 countries and territories from 1990 to 2015. It covers 310 diseases and injuries, 2,619 sequelae and 79 risk factors by age and sex. Detailed descriptions of the methodology and approach of GBD 2015 have been published elsewhere [9,10].

### Colorectal cancer estimates

We mapped all neoplasms as defined by the 10th revision of the International Statistical Classification of Diseases (ICD-10) to one of the 29 GBD cancer groups. ICD-10 codes for mortality by colorectal cancer in GBD study were C18-C21.9, D01.0-D01.3, D12-D12.9, D37.3-D37.5 [3, 9, 11].

Input data for cancer mortality estimates came from Vital registry mortality and Cancer registry incidence data. Most cancer registries only report cancer incidence. However, if a cancer registry also reported cancer mortality, mortality data were also extracted from the source to be used in the mortality to incidence estimation. In the case when high quality mortality data were available but not reported by the registry, processed (post-redistribution) vital registration mortality data from the cause of death database were matched to the registry’s incidence data [3, 9, 11]. The accuracy of mortality data reported in cancer registries usually depends on the quality of the vital registration system. If the vital registration system is incomplete or of poor quality the mortality to incidence ratio can be biased to lower ratios. In this study, data were excluded if they were not representative of the coverage population (e.g., hospital based registries), if they did not cover all malignant neoplasms as defined in ICD9 (140–208) or ICD10 (C00-C96) (e.g., specialty cancer registry), if they did not include data for both sexes and all age groups, if the data were limited to years prior to 1980, or if the source did not provide details on the population covered. Preference was given to registries with national coverage over those with only local coverage, except those from countries where the GBD study provides sub-national estimates, as is the case in Brazil [3, 9, 11]. Studies of demography and statistics [12, 13] showed that there is a considerable improvement in the completeness of the death-count coverage in Brazil since 1980. In the Southeast and Southern Brazil there is complete coverage of the adult mortality registry. In the Northeast and North Brazil, there were still places with a low coverage, although there was a clear improvement in the quality of data. For all Brazilian states, the quality of the data from the vital and cancer registries is considered high and close to high-income countries [12, 13]. Cancer registry incidence data were transformed to mortality estimates using separately modeled mortality-to incidence ratios (MIR) [11]. Multiple logit random effect models were created. All models were run and the best model was selected. All models were tested at multiple stages before creating the final model output. The best model was selected based on the lowest mean out-of sample Root-Mean-Squared Error (RMSE) from those models remaining after checking the mean MIR. All of the modeling details have been published previously [3, 9, 11].

The raw data were processed to make them comparable and to account for ‘‘garbage codes”, which are codes assigned to causes that are not usable from a public health perspective [14]. These causes were redistributed to the most likely underlying cause of death based on a regression model [3]. Using a cause of death ensemble modeling (CODEm) approach with cause-specific covariates, we computed mortality estimates for each individual cause [15].

Cancer survival was calculated using a MIR-based scaling factor. We calculated 10-year prevalence of each cancer and each incidence cohort using these cancer survival estimates. The total prevalence was divided into four sequelae with variable disability weights: (1) diagnosis and treatment, (2) remission, (3) metastatic, and (4) terminal phase. We assumed a constant duration for sequelae (1), (3), and (4). Duration of sequela (2) was the remaining prevalence after subtracting the duration of the fixed sequelae. We computed years of life lost (YLLs) by multiplying deaths by the normative standard life expectancy at each age of death. For each sequela, Years Lost due to Disability (YLDs) were calculated by multiplying the prevalence of each sequela by its disability weight. Finally, DALYs were calculated by summing premature death (YLLs) and YLDs. More information about these estimates can be found elsewhere [9].

### Physical inactivity estimate

Surveys of the general adult population, performed using random sampling procedures, were included that captured self-reported physical activity in all domains of life (leisure/recreation, work, household and transport). Due to the absence of a consistent relationship, on the individual level, between the amount of activity performed in each domain and total activity, it was not possible to use studies that included only recreational/leisure activities [16].

For the global estimates, data were primarily derived from two standardized questionnaires, The Global Physical Activity Questionnaire and the International Physical Activity Questionnaire, although any other survey instrument was included that asked about the intensity, frequency and duration of physical activities performed across all activity domains [16].

In the case of Brazil [7], we consulted existing surveys such the Telephone-based Surveillance of Risk and Protective Factors for Chronic Diseases, Brazil World Health Survey, and the International Prevalence Study on Physical Activity. More details can be found at http://ghdx.healthdata.org/gbd-2015/data-input-sources.

To standardize all estimates in Brazil and around the world, we considered data from the population aged 25 years or more. The physical activity was accumulated for durations of at least ten consecutive minutes, across all domains of life. The frequency, duration and intensity of activity were used to calculate the total metabolic equivalent (MET) minutes per week. The estimates were made for the subjects classified as physically inactive (<600 METS-min/week) [16].

### Analytic methods

The contribution of physical inactivity in the mortality and DALYs by colorectal cancer was estimated using a comparative risk assessment approach in which observed health outcomes are compared to those that would have been observed with a counterfactual set of exposure where no one is exposed [16]. For this, we used the Cause of Death Ensemble Modeling-CODEm (CODEm) that is used to estimate indicators by age, sex, country, year, and cause, and is an analytical tool that tests several possible statistical models of causes of death and creates a combined set of models that offers the best predictive performance. The software DisMod-MR 2.1, a meta-regression tool, is used for simultaneous estimates of incidence, prevalence, remission, disability, and also mortality, attributable to risk factors, such as physical inactivity [16]. Modeling details can be found in the literature [9, 11, 16].

In this study, absolute numbers of deaths, the mortality rates and DALYs (per 100,000 inhabitants—crude and age-standardized) were used as the metric. The sum of DALYs across the population, or the burden of disease, can be thought of as a measurement of the gap between current health status and an ideal health situation [17]. The standard population used in this study in the corresponding age groups was estimated population from the World Population Prospect 2015 Revision by the United Nations Population Division [18]. For subnational locations, as is the case in Brazil, interpolation and extrapolation based on rate of change are used together with age specific population from censuses. Raking was applied to ensure consistency between subnational and national populations [9].

In the GBD study, 95% uncertainty intervals (95%U.I.) were calculated, to provide information on the variability of estimates resulting from errors due to the sampling process, and also non-sample errors due to adjustments of data sources and modeling [9].

The GBD 2015 created the Socioeconomic Development Index (SDI) [3, 9, 11] for all evaluated locations, by calculating per capita income, formal education at 15 years of age and fertility rate. In the present study, this index was used to compare the metrics used among the states of Brazil. For this, the Spearman correlation coefficient was applied. In all analyzes between colorectal cancer and physical inactivity, the population considered was aged ≥ 25 years. For the supplementary tables that present only the estimates for colorectal cancer we consider the entire population (i.e. ≥ 15 years old).

## Results

Around the world, 487,860 deaths by colorectal cancer were estimated in 1990 and 832,048 in 2015. In Brazil, 6,894 deaths from colorectal cancer were estimated in 1990 and 21,419 in 2015. From 1990 to 2015, mortality from colorectal cancer increased more in Brazil than in the rest of the world (S1 Dataset and S1 Table).

Around the world, 10,777,181 DALYs for colorectal cancer were estimated in 1990 and 17,026,563 in 2015. In Brazil, 171,831 DALYs were estimated in 1990 and 467,421 in 2015. From 1990 to 2015, DALYs from colorectal cancer increased more in Brazil than in the rest of the world (S2 Table).

Regarding the mortality by physical inactivity due to all causes, 1,031,823 deaths were estimated in 1990 and 1,605,494 in 2015 around the world. In Brazil, 33,227 deaths were estimated in 1990 and 59,197 in 2015. From 1990 to 2015, mortality attributed to physical inactivity from all causes increased 33.6% (95%U.I.: 30.3–38.0) around the world and 19.6% (95%U.I.: 15.0–25.6) in Brazil (S3 Table).

Around the world, 22,318,505 DALYs from physical inactivity due to all causes were estimated in 1990 and 34,603,468 in 2015. In Brazil, 863,721 DALYs were estimated in 1990 and 1,401,966 in 2015. From 1990 to 2015, DALYs from physical inactivity due to all causes increased around the world, but did not increase in Brazil (S4 Table).

In relation to colorectal cancer mortality due to physical inactivity, 69,456 deaths were estimated in 1990 and 119,351 in 2015 around the world. These values represented, in 1990, an age-standardized rate of mortality of 2.1 (95%U.I.: 1.5–2.8) and of 1.9 (95%U.I: 1.3–2.4) in 2015. In Brazil, 1,302 deaths were estimated in 1990 and 4,143 in 2015 that represented, in 1990, an age-standardized rate of mortality of 2.0 (95%U.I: 1.4–2.5) and of 2.4 (95%U.I: 1.8–3.0) in 2015. From 1990 to 2015, the mortality by colorectal cancer due to physical inactivity increased in Brazil (0.6%; 95%U.I: 0.1–1.4) and decreased around the world (-0.8%; 95%U.I.: -1.5 - -0.2). The Brazilian state with the greatest increase (1990–2015) in colorectal cancer mortality due to physical inactivity was São Paulo (Table 1).

**Table 1:** Number and age-standardized rate (per 100,000 inhabitants) of deaths from colorectal cancer due to physical inactivity globally, in Brazil, and in the Brazilian states.

Deaths due to colorectal cancer attributable to physical inactivity															
	1990			2015			1990			2015			Change (1990–2015)		
	Deaths	95% U.I.		Deaths	95% U.I.		Rate*	95% U.I.		Rate*	95% U.I.		%*	95% U.I.	
Global	69,456	48,533	90,67	119,351	83,913	155,791	2.15	1.51	2.80	1.90	1.34	2.47	-0.84	-1.54	-0.24
Brazil	1,302	966	1,639	4,143	3,099	5,189	2.00	1.49	2.50	2.41	1.81	3.02	0.66	0.15	1.41
Acre	02	01	02	06	05	08	1.18	0.88	1.50	1.58	1.17	2.03	0.65	-0.08	1.43
Alagoas	12	09	15	33	24	43	1.19	0.88	1.50	1.48	1.11	1.93	0.22	-0.75	1.25
Amapá	01	01	01	04	03	06	0.88	0.65	1.13	1.36	0.95	1.81	0.59	-0.36	1.59
Amazonas	09	07	11	36	25	47	1.55	1.15	1.98	1.95	1.37	2.58	0.52	-0.39	1.61
Bahia	78	58	99	238	174	308	1.56	1.18	1.99	2.02	1.48	2.61	0.25	-0.67	1.22
Ceará	34	24	43	129	93	167	1.15	0.83	1.46	1.86	1.35	2.40	0.30	-0.49	1.26
Distrito Federal	10	07	12	50	36	65	2.09	1.55	2.66	2.36	1.69	3.09	0.77	-0.12	1.95
Espírito Santo	19	13	24	68	50	89	1.75	1.27	2.22	2.06	1.51	2.68	0.68	-0.21	1.97
Goiás	27	20	34	107	77	138	1.88	1.40	2.35	2.20	1.59	2.83	0.42	-0.61	1.62
Maranhão	27	19	35	65	45	89	1.38	0.98	1.80	1.52	1.07	2.06	0.88	-0.05	2.13
Mato Grosso	09	07	12	43	31	55	1.60	1.20	2.04	2.03	1.48	2.64	0.69	-0.37	1.96
Mato Grosso do Sul	11	08	15	45	32	60	1.71	1.26	2.21	2.25	1.62	2.96	0.12	-0.97	1.42
Minas Gerais	126	93	159	430	315	553	1.79	1.32	2.26	2.18	1.59	2.80	-0.07	-1.06	0.93
Paraná	76	55	97	271	197	352	2.19	1.60	2.77	2.71	1.97	3.50	0.51	-0.49	1.75
Paraíba	19	14	24	54	38	71	1.15	0.84	1.47	1.63	1.16	2.14	0.17	-0.60	1.05
Pará	21	15	27	73	51	97	1.32	0.96	1.68	1.65	1.17	2.16	0.44	-0.55	1.71
Pernambuco	44	33	57	120	86	158	1.28	0.95	1.64	1.67	1.21	2.19	0.85	0.03	2.01
Piaui	12	08	15	35	25	46	1.14	0.83	1.46	1.50	1.08	1.94	0.21	-0.71	1.30
Rio de Janeiro	186	138	236	508	369	654	2.61	1.94	3.33	2.94	2.15	3.79	0.49	-0.31	1.52
Rio Grande do Norte	15	11	19	46	33	61	1.26	0.95	1.60	1.69	1.20	2.22	0.20	-0.87	1.50
Rio Grande do Sul	141	103	178	369	264	490	2.93	2.15	3.71	2.98	2.13	3.95	0.61	-0.41	1.88
Rondônia	04	03	05	15	11	20	1.38	1.02	1.74	1.54	1.13	1.99	0.73	-0.03	1.59
Roraima	00	00	01	03	02	04	1.05	0.79	1.31	1.34	0.99	1.75	0.82	0.02	1.81
Santa Catarina	39	28	49	136	96	175	2.17	1.61	2.78	2.36	1.69	3.03	0.61	-0.39	1.90
Sergipe	08	06	11	25	18	33	1.27	0.93	1.64	1.67	1.19	2.19	0.57	-0.42	1.78
São Paulo	370	272	470	1,218	891	1,574	2.51	1.86	3.19	2.96	2.17	3.82	0.90	0.01	2.07
Tocantins	03	02	04	15	11	20	1.06	0.73	1.41	1.59	1.13	2.11	0.76	-0.28	2.03

*Age-standardized rate; U.I.: uncertainty interval

In relation to colorectal cancer DALYs due to physical inactivity, 1,391,037 DALYs were estimated in 1990 and 2,209,209 in 2015 around the world. These values represented, in 1990, an age-standardized rate of mortality of 38.8 (95%U.I.: 26.7–51.3) and of 33.2 (95%U.I: 23.0–43.6) in 2015. In Brazil, 31,121 DALYs were estimated in 1990 and 87,116 in 2015 that represented, in 1990, an age-standardized rate of mortality of 38.3 (95%U.I: 28.3–48.2) and of 45.7 (95%U.I: 34.0–57.3) in 2015. From 1990 to 2015, the DALYs by colorectal cancer due to physical inactivity increased in Brazil (0.6%; 95%U.I: 0.1–1.4) and decreased around the world (-1.1%; 95%U.I.: -2.0 - -0.3). The Brazilian state with the greatest increase (1990–2015) in colorectal cancer DALYs due to physical inactivity was São Paulo (Table 2).

**Table 2:** Number and age-standardized rate (per 100,000 inhabitants) of deaths from colorectal cancer due to physical inactivity globally, in Brazil, and in the Brazilian states.

DALYs due to colorectal cancer attributable to physical inactivity															
	1990			2015			1990			2015			Change (1990–2015)		
	DALYs	95% U.I.		DALYs	95% U.I.		Rate*	95% U.I.		Rate*	95% U.I.		%*	95% U.I.	
Global	1,391,037	952,242	1,850,921	2,209,209	1,528,502	2,915,924	38.86	26.74	51.37	33.20	23.06	43.69	-1.10	-2.05	-0.36
Brazil	31,121	22,933	39,367	87,116	64,619	109,15	38.34	28.36	48.27	45.77	34.03	57.32	0.68	0.11	1.48
Acre	40	29	51	145	107	190	22.84	16.81	28.80	30.48	22.66	39.74	0.49	-0.39	1.48
Alagoas	288	212	367	763	551	991	23.59	17.44	29.87	29.63	21.52	38.32	-0.13	-1.34	1.13
Amapá	18	13	23	106	74	146	16.61	12.16	21.30	25.93	18.08	35.00	0.48	-0.57	1.59
Amazonas	234	173	301	881	604	1,169	30.15	22.40	38.62	38.07	26.40	50.38	0.21	-0.87	1.42
Bahia	1,744	1,306	2,21	5,07	3,643	6,585	29.44	22.07	37.26	38.75	27.91	50.23	0.17	-1.01	1.29
Ceará	759	544	969	2,760	2,004	3,572	21.80	15.76	27.73	36.58	26.53	47.22	0.44	-0.44	1.44
Distrito Federal	269	192	346	1,011	718	1,328	39.27	28.61	50.09	41.80	29.70	54.82	0.56	-0.41	1.71
Espírito Santo	456	323	586	1,447	1,038	1,918	33.41	23.85	42.49	39.08	28.17	51.53	0.47	-0.57	1.87
Goiás	713	527	903	2,458	1,739	3,165	36.94	27.44	46.57	42.80	30.57	54.93	0.13	-1.05	1.46
Maranhão	657	463	867	1,483	1,012	2,059	28.05	19.85	36.99	30.73	21.10	42.64	0.66	-0.43	1.96
Mato Grosso	250	184	321	1,008	725	1,320	30.78	22.92	39.03	38.93	28.13	50.90	0.68	-0.67	2.22
Mato Grosso do Sul	288	207	373	986	693	1,3	32.82	23.69	42.45	42.81	30.30	56.21	-0.16	-1.39	1.18
Minas Gerais	2,994	2,176	3,825	9,046	6,542	11,817	34.06	25.06	43.26	42.44	30.76	55.29	-0.36	-1.41	0.62
Paraná	1,874	1,342	2,382	5,758	4,116	7,559	41.72	30.10	52.93	51.75	37.14	67.56	0.38	-0.75	1.63
Paraíba	426	309	543	1,159	816	1,553	22.49	16.34	28.53	32.55	22.93	43.37	0.13	-0.76	1.09
Pará	531	382	684	1,746	1,205	2,334	25.42	18.44	32.67	32.22	22.38	42.86	0.29	-0.85	1.61
Pernambuco	1,059	776	1,371	2,655	1,886	3,528	25.76	18.87	33.25	32.89	23.44	43.54	0.72	-0.27	1.96
Piaui	277	199	358	792	558	1,041	22.15	16.01	28.34	29.72	21.06	38.89	0.20	-0.96	1.48
Rio de Janeiro	4,498	3,330	5,718	10,379	7,526	13,368	50.85	37.54	64.36	56.55	41.05	72.92	0.81	-0.12	2.03
Rio Grande do Norte	312	228	397	973	689	1,286	23.25	17.23	29.53	32.38	22.98	42.60	0.10	-1.05	1.47
Rio Grande do Sul	3,220	2,328	4,085	7,299	5,141	9,769	54.51	39.71	68.91	55.93	39.34	74.61	0.42	-0.67	1.70
Rondônia	112	82	142	376	263	491	27.61	20.25	34.91	29.73	21.26	38.32	0.71	-0.17	1.72
Roraima	13	10	17	69	50	89	20.14	15.11	25.28	24.92	18.33	32.28	0.82	-0.10	2.05
Santa Catarina	920	671	1,178	2,849	1,989	3,715	40.21	29.62	50.94	43.48	30.67	56.68	0.18	-0.89	1.47
Sergipe	188	135	244	578	403	773	24.82	18.01	32.08	32.99	23.26	43.91	0.54	-0.52	1.77
São Paulo	8,900	6,460	11,434	24,980	18,051	32,107	47.64	34.95	60.55	55.16	40.07	70.88	1.11	0.20	2.42
Tocantins	82	55	111	337	238	448	20.37	13.94	27.41	30.54	21.63	40.42	0.74	-0.46	2.14

*Age-standardized rate; U.I.: uncertainty interval

Mortality and DALYs due to colorectal cancer due to physical inactivity in Brazil increased with increasing age in 1990 and 2015. Table 3 shows the information according to age for absolute number and age-standardized rate of deaths and DALYs.

**Table 3:** Absolute number and rate non-standardized of death and DALYs from colorectal cancer due to all causes and attributed to physical inactivity in Brazil in 1990 and 2015 by age.

	Deaths due to colorectal cancer attributable to physical inactivity									DALYs due to colorectal cancer attributable to physical inactivity								
	15–49 years			50–69 years			70+ years			15–49 years			50–69 years			70+ years		
	Metric	95% U.I.		Metric	95% U.I.		Metric	95% U.I.		Metric	95% U.I.		Metric	95% U.I.			95% U.I.	
Males (1990)																		
Number	84	60	108	263	195	335	243	182	306	3,965	2,868	5,104	7,222	5,371	9,213	3,326	2,484	4,188
Rate*	0.22	0.16	0.28	3.54	2.63	4.51	17.12	12.85	21.57	10.24	7.41	13.18	97.06	72.17	123.80	234.18	174.94	294.86
Males (2015)																		
Number	192	139	248	798	585	1,025	1,004	745	1,264	8,954	6,471	11,565	22,166	16,302	28,361	12,252	9,121	15,557
Rate*	0.34	0.25	0.44	4.65	3.42	5.98	23.63	17.54	29.75	15.88	11.47	20.51	129.28	95.08	165.41	288.34	214.64	366.13
Female (1990)																		
Number	99	72	125	282	206	357	329	246	414	4,668	3,418	5,926	7,786	5,679	9,841	4,151	3,086	5,231
Rate*	0.25	0.18	0.32	3.44	2.51	4.34	15.80	11.82	19.88	11.88	8.70	15.08	94.63	69.02	119.60	199.03	148.03	250.88
Female (2015)																		
Number	212	155	273	749	546	945	1,186	891	1,488	12,872	9,631	16,065	9,837	7,215	12,621	21,033	15,352	26,505
Rate*	0.37	0.27	0.48	3.87	2.82	4.88	19.50	14.63	24.46	17.34	12.72	22.24	108.67	79.32	136.94	211.57	158.31	264.07

* non-standardized rate (per 100,000 inhabitants)

**Fig 1:**
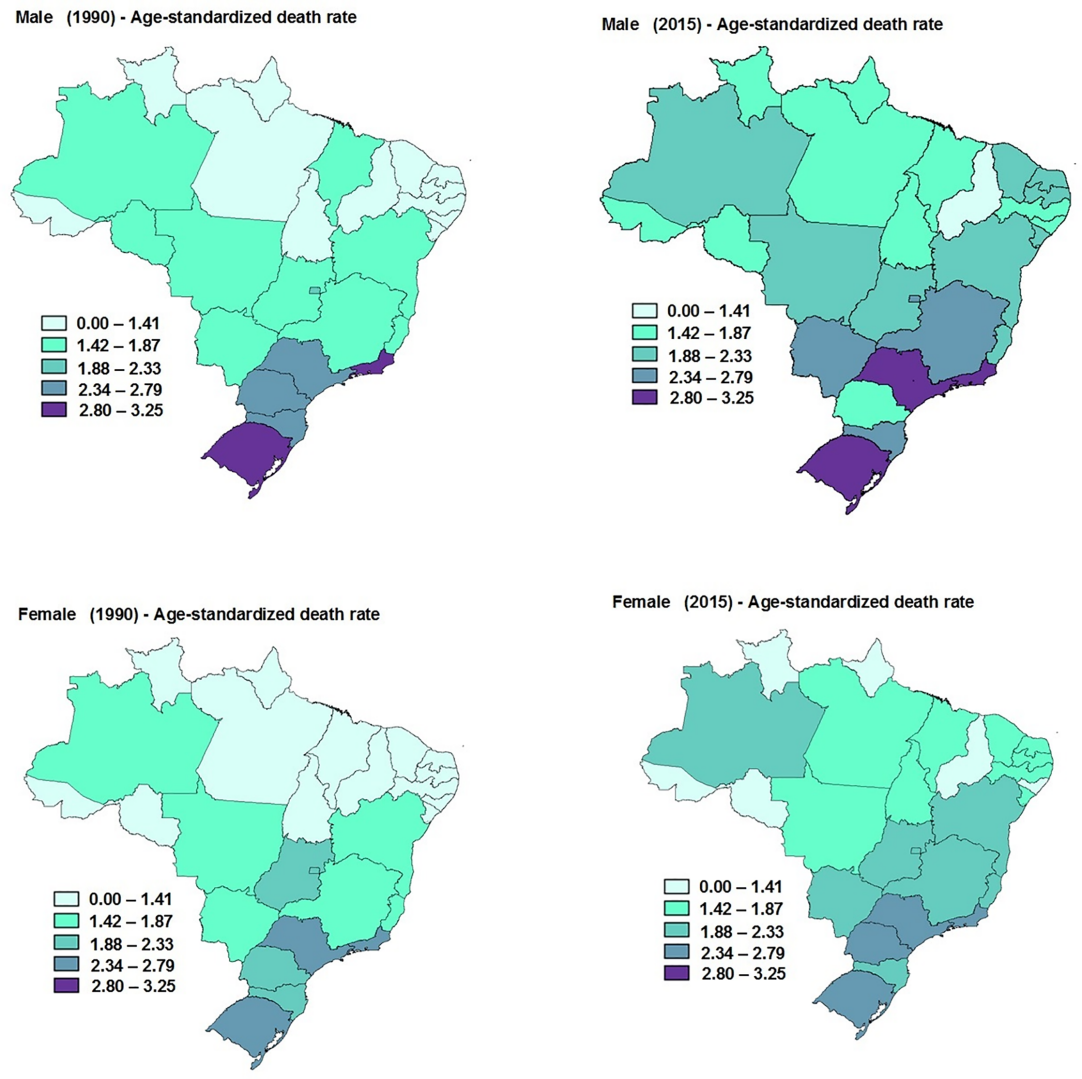
Age-standardized death rate (per 100,000 inhabitants) from colorectal cancer due to physical inactivity in males and females in the Brazilian states.

The age-standardized rate of mortality by colorectal cancer due to physical inactivity was similar between men and women in Brazil, both in 1990 (men: 2.0, 95%U.I.: 1.5–2.6; women: 1.9, 95%U.I.: 1.4–2.4) and 2015 (men: 2.7, 95%U.I: 2.0–3.4; women: 2.1, 95%U.I.: 1.6–2.7). From 1990 to 2015, colorectal cancer mortality rates due to physical inactivity did not change significantly among Brazilian states when analyses were stratified by sex (Fig 1).

**Fig 2:**
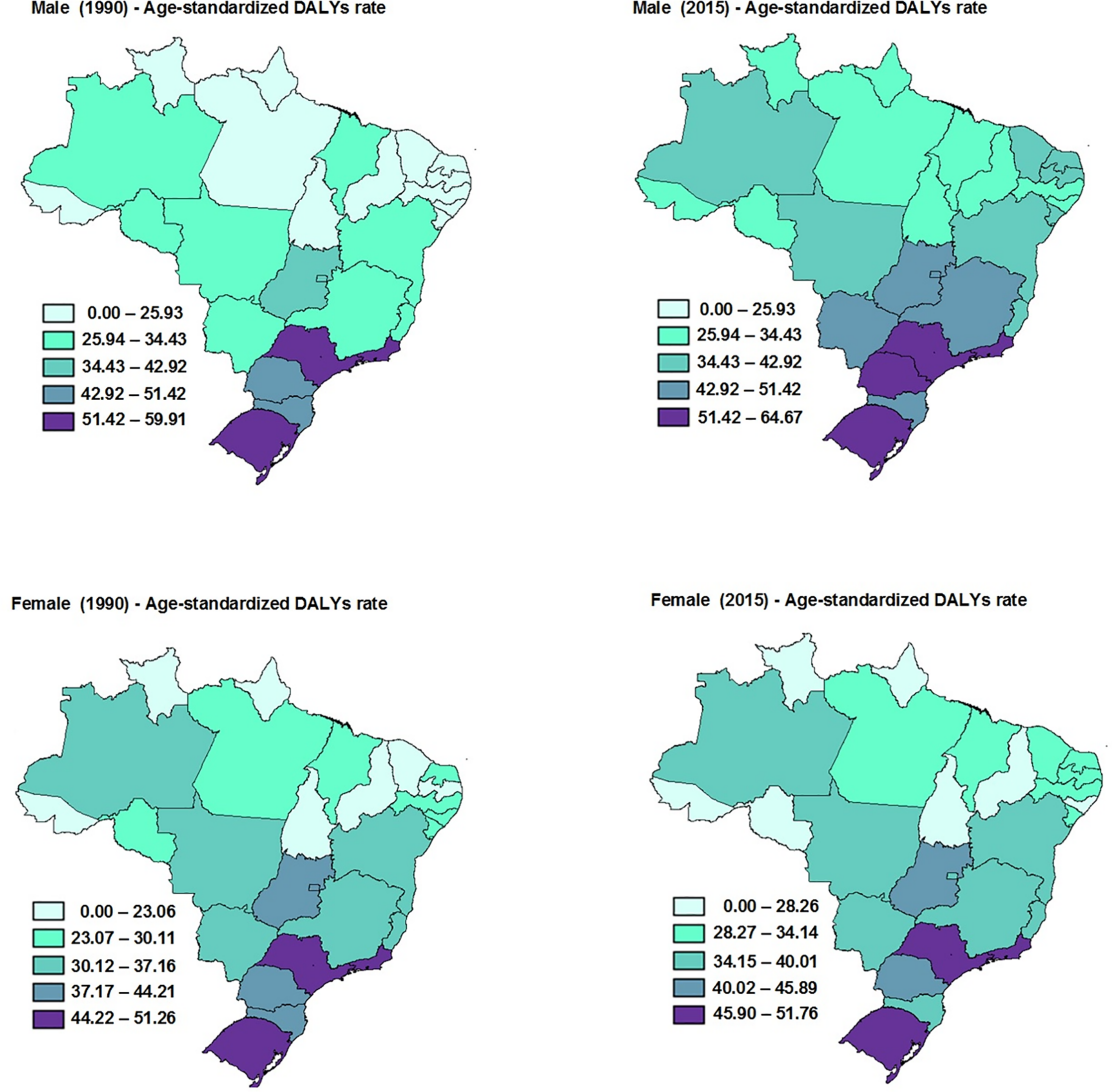
Age-standardized DALYs (per 100,000 inhabitants) from colorectal cancer due to physical inactivity in males and females in the Brazilian states.

The age-standardized rate of DALYs by colorectal cancer due to physical inactivity was similar between men and women in Brazil, both in 1990 (men: 39.7, 95%U.I.: 29.6–50.3; women: 37.4, 95%U.I.: 27.4–47.1) and 2015 (men: 50.9, 95%U.I: 37.8–64.7; women: 41.8, 95%U.I.: 31.0–52.3). When the analyses were stratified by Brazilian state, men from São Paulo presented an increase age-standardized rate of DALYs from 1990 to 2015 (1.5%; 95%U.I.: 0.3–3.3), but for women there was no increase. In the other states of Brazil there were no significant changes in age-standardized rate of DALYs from 1990 to 2015 (Fig 2).

**Fig 3:**
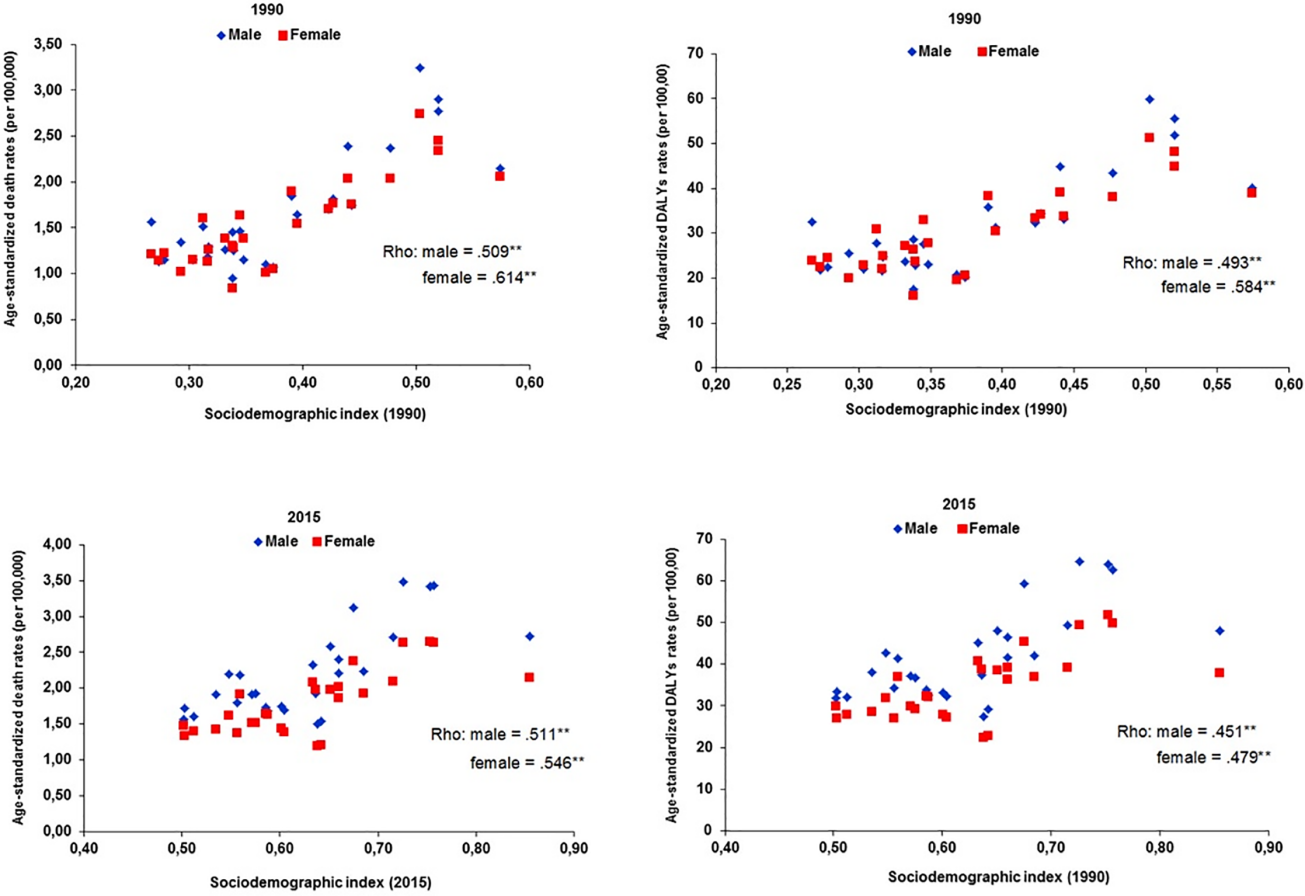
Relationship between age-standardized rate and DALYs from colorectal cancer due to physical inactivity and sociodemographic index of the Brazilian states in 1990 and 2015 according to sex. Rho: Spearman's correlation coefficient; ** p <0.01.

The states of Brazil with better socioeconomic indicators had higher mortality rates and DALYs by colorectal cancer due to physical inactivity (p<0.01). The magnitudes of association between mortality and DALYs rates with SDI were relatively higher for women than for men (Fig 3).

## Discussion

The main finding of this study was that in Brazil, the growth of colorectal cancer mortality rates, between 1990 and 2015, was higher than the overall global rates (68.47% *vs* 24.56%). Physical inactivity was responsible for 1,302 deaths in 1990 and 4,143 in 2015 due to colorectal cancer. For DALYs, the growth of colorectal cancer rates was higher in Brazil (67.19%) than overall global rates (22.92%), and physical inactivity was responsible for 31,121 cases in 1990 and 87,116 cases in 2015 due to colorectal cancer. In addition, there were more worrisome estimates for Brazil compared to around the world, because in Brazil there was an increase in mortality (0.66%) and DALYs (0.68%) rates by colorectal cancer due to physical inactivity over 25 years (1990–2015) and globally there was a decline (deaths: -0.84%; DALYs: -1.10%). This information reinforces physical inactivity is an independent risk factor for colorectal cancer and raises particular cause for concern in Brazil. One of the possible explanations for this increase observed in the present study may be the fact that in this period the risk of exposure to physical inactivity in adults in Brazil was 70% and around the world was 40% [7]. That is, in Brazil, the population has a higher risk of exposure to physical inactivity.

Regular physical activity is known to have a protective effect against colorectal cancer [19, 20], even in individuals with different values of body mass index [21], for different domains of physical activity [19,20] and after controlling for many lifestyle factors [21]. However, there are few studies on the influence of socioeconomic level in relation to physical inactivity and colorectal cancer. Analyses of mortality and DALYs according to socioeconomic status of the city are provided in the GBD study and identify iniquities in health [9]. In this study we observed that in the Brazilian states with better socioeconomic conditions the mortality and DALYs rates from colorectal cancer due to physical inactivity were higher in comparison with the states of worst socioeconomic conditions. This result is contrary to that presented to the United States in a study that analyzed mortality from colorectal cancer due to all causes for a period of 30 years [22]. The authors reported that in the 1980s, colorectal cancer mortality was more prominent in the population with a high socioeconomic level. However, from the mid-1990s this was reversed, and higher mortality rates were observed in the population with lower socioeconomic status. According to the authors, this was due to advances in medical procedures and the early detection of colorectal cancer being more accessible to the population with a high socioeconomic level [22].

Evidence exists that reductions in colorectal cancer mortality can be achieved through detection and treatment of early-stage colorectal cancer and the identification and removal of adenomatous polyps, the precursor to these cancers [23]. In the WHO Noncommunicable Diseases Plan, countries were recommended to implement population-based colorectal cancer screening, including using a fecal occult blood test as appropriate) at age >50, linked with timely treatment [23]. However, this measure, which could lead to the detection and reduction of mortality, was not implemented in Brazil. It is necessary to broaden this discussion and accelerate measures for greater population prevention and early treatment of colorectal cancer.

Mortality and DALYs rates for colorectal cancer due to physical inactivity were similar between men and women from Brazil. When we analyzed the trend from 1990 to 2015 an increase in mortality and DALYs rates in both sexes was observed. There are no other studies that analyzed the mortality and DALYs trend by colorectal cancer due to physical inactivity, which does not allow us to make comparisons with other countries. We expected that men had higher mortality and higher DALYs than in women because the prevalence of sufficient physical activity during leisure time among Brazilian women increased more than in men over the last decade [24,25], and this may result in a decrease in morbidity and mortality by colorectal cancer. In addition to this, women more frequently participate in preventive tests compared to men [26,27]. In Brazil, for example, this situation resulted in the Men's Health Policy which aims to show men the importance of preventive health examinations and of the healthy lifestyle throughout life [27]. However, our hypothesis has not been confirmed and for Brazil more actions are necessary to stimulate the practice of physical activity and preventive tests for colorectal cancer for the whole population.

This present study has important limitations worth noting. The analysis of one type of cancer does not allow inferences to other types of cancer where physical inactivity is considered a risk factor, such as breast cancer [5]. Non-distinction of the tumor site in the colon (proximal, distal, or other) may be considered a limitation because it did not allow us to identify for which of these sites the mortality or DALYs were higher, as done in other studies [2]. Also, the present study only includes physical activity measured by questionnaires which are considered subjective measures of physical activity and associated with measurement bias [28]. The non-stratification of physical activity by domains is another limitation.

The strength of this study was to work with mortality and physical inactivity information from all over Brazil. From this information it was possible to estimate mortality rates and DALYs by colorectal cancer due to physical inactivity. Until now it was known that physical inactivity was a risk factor for colorectal cancer, but it had not been estimated how much it caused of morbidity (DALYs) and mortality, especially in a trend analysis of 25 years.

It can be concluded that physical inactivity was responsible for a substantial number of deaths and DALYs due to colorectal cancer in Brazil. Estimates of mortality and morbidity from colorectal cancer as a result of physical inactivity in Brazil increased between 1990 and 2015 compared to global estimates. The Brazilian states with better socioeconomic indicators had higher rates of mortality and morbidity by colorectal cancer due to physical inactivity.

## Supporting information

S1 Dataset (XLSX)

S1 Table This is the S1 number and age-standardized death (per 100,000 inhabitants) of deaths from colorectal cancer due to all causes globally, in Brazil, and in the Brazilian states. *Age-standardized rate; U.I.: uncertainty interval.
(PDF)

S2 Table This is the S2 number and age-standardized rate (per 100,000 inhabitants) of DALYs from colorectal cancer due to all causes globally, in Brazil, and in the Brazilian states. *Age-standardized rate; U.I.: uncertainty interval.
(PDF)

S3 Table This is the S3 number and age-standardized rate (per 100,000 inhabitants) of deaths from physical inactivity due to all causes globally, in Brazil, and in the Brazilian states. *Age-standardized rate; U.I.: uncertainty interval.
(PDF)

S4 Table This is the S4 number and age-standardized rate (per 100,000 inhabitants) of DALYs from physical inactivity due to all causes globally, in Brazil, and in the Brazilian states. *Age-standardized rate; U.I.: uncertainty interval.
(PDF)
