# Supplementary material for: Mortality and years of life lost by colorectal cancer attributable to physical inactivity in Brazil (1990–2015): Findings from the Global Burden of Disease Study
Source: PLoS One. 2018 Feb 1;13(2):e0190943. doi: 10.1371/journal.pone.0190943 (PMC5794056; doi:10.1371/journal.pone.0190943)
Supplement: S1 Table — *Age-standardized rate; U.I.: uncertainty interval. (PDF) [file pone.0190943.s002.pdf]

Supplementary File 1.

Number and age-standardized death (per 100,000 inhabitants) of deaths from colorectal cancer due to all causes globally, in Brazil, and in the Brazilian states.

Mortality by Colorectal cancer due to all causes														
	1990			2015			1990			2015			Change (1990-2015)	
	Deaths	95% U.I.		Deaths	95% U.I.		Rate*	95% U.I.		Rate*	95% U.I.		%*	95% U.I.	
Global	487,860	479,468	496,479	832,048	811,656	854,532	14.65	14.40	14.92	13.04	12.72	13.40	24.56	20.79	28.19
Brazil	6,894	6,688	7,121	21,419	20,140	22,725	10.28	9.96	10.63	12.36	11.61	13.16	68.47	60.80	76.98
Acre	08	07	09	30	26	36	5.77	5.17	6.41	7.67	6.55	8.95	65.04	46.49	86.06
Alagoas	61	55	67	166	143	191	5.90	5.30	6.54	7.36	6.39	8.46	77.70	59.56	98.45
Amapá	04	03	04	22	17	27	4.38	3.85	4.95	6.71	5.39	8.21	64.24	42.76	89.12
Amazonas	45	40	51	178	149	212	7.61	6.75	8.54	9.57	8.01	11.29	58.15	40.54	77.29
Bahia	400	360	440	1,211	1,035	1,406	7.88	7.12	8.65	10.19	8.72	11.82	59.00	42.17	76.33
Ceará	173	153	193	654	561	757	5.79	5.12	6.48	9.37	8.03	10.87	86.82	63.07	113.93
Distrito Federal	53	49	57	260	224	300	10.85	10.00	11.87	12.13	10.44	14.03	71.64	52.49	94.26
Espírito Santo	101	92	110	357	306	409	9.11	8.31	9.96	10.65	9.17	12.16	62.58	46.84	80.13
Goiás	143	131	155	546	476	628	9.45	8.64	10.32	11.01	9.64	12.60	61.69	45.25	81.11
Maranhão	140	120	163	332	271	408	7.02	6.05	8.23	7.67	6.28	9.36	50.27	28.74	72.42
Mato Grosso	48	43	53	216	181	254	7.97	7.11	8.87	10.09	8.50	11.76	64.00	46.42	83.55
Mato Grosso do Sul	62	56	68	241	207	282	8.98	8.14	9.91	11.82	10.15	13.78	69.50	52.53	89.27
Minas Gerais	672	616	732	2,256	1,951	2,604	9.30	8.47	10.19	11.35	9.85	13.04	78.02	60.07	97.26
Paraná	412	378	449	1,423	1,234	1,639	11.42	10.48	12.53	14.04	12.18	16.14	71.54	54.89	91.10
Paraíba	96	86	107	266	221	320	5.68	5.09	6.29	8.02	6.67	9.63	60.38	40.59	80.98
Pará	111	98	125	378	312	457	6.67	5.87	7.49	8.32	6.95	9.97	54.92	37.26	75.72
Pernambuco	233	210	259	618	530	722	6.59	5.97	7.29	8.51	7.31	9.93	74.61	56.62	95.58
Piaui	60	52	69	178	151	208	5.69	4.91	6.53	7.50	6.39	8.67	53.44	30.92	78.92
Rio de Janeiro	969	902	1,044	2,580	2,276	2,948	13.25	12.29	14.32	14.86	13.10	17.00	69.34	53.12	86.23
Rio Grande do Norte	77	70	84	240	206	279	6.51	5.91	7.15	8.67	7.43	10.08	61.37	42.09	83.00
Rio Grande do Sul	757	690	827	1,928	1,646	2,276	15.37	14.01	16.80	15.49	13.22	18.28	41.66	27.12	57.34
Rondônia	19	17	21	74	62	85	6.61	5.96	7.31	7.32	6.26	8.41	64.07	48.04	80.82
Roraima	2	2	3	14	12	16	5.16	4.71	5.64	6.53	5.63	7.52	72.99	54.70	91.80
Santa Catarina	206	188	226	706	603	822	11.22	10.24	12.32	12.11	10.39	14.05	59.50	44.40	76.84
Sergipe	44	39	48	132	112	155	6.62	5.93	7.29	8.66	7.40	10.06	63.05	45.28	83.41
São Paulo	1,982	1,842	2,130	6,336	5,481	7,193	13.06	12.04	14.12	15.25	13.19	17.34	77.51	60.35	96.84
Tocantins	18	15	21	78	63	92	5.44	4.54	6.58	8.13	6.76	9.56	71.95	44.65	104.62

*Age-standardized rate; U.I.: uncertainty interval
