# Supplementary material for: Mortality and years of life lost by colorectal cancer attributable to physical inactivity in Brazil (1990–2015): Findings from the Global Burden of Disease Study
Source: PLoS One. 2018 Feb 1;13(2):e0190943. doi: 10.1371/journal.pone.0190943 (PMC5794056; doi:10.1371/journal.pone.0190943)
Supplement: S2 Table — *Age-standardized rate; U.I.: uncertainty interval. (PDF) [file pone.0190943.s003.pdf]

Supplementary File 2.

Number and age-standardized rate (per 100,000 inhabitants) of DALYs from colorectal cancer due to all causes globally, in Brazil, and in the Brazilian states.

DALYs by Colorectal cancer due to all causes															
	1990			2015			1990			2015			Change (1990-2015)		
	DALYs	95% U.I.		DALYs	95% U.I.		Rate*	95% U.I.		Rate*	95% U.I.		%*	95% U.I.	
Global	10,777,181	10,553,336	10,994,209	17,026,563	16,586,696	17,504,661	290.88	285.04	296.77	251.35	244.92	258.39	22.92	18.48	27.08
Brazil	171,831	166,310	177,585	467,421	442,829	494,577	205.14	198.73	211.90	243.21	230.53	257.29	67.19	58.65	76.56
Acre	210	188	234	749	636	879	115.11	103.77	127.69	152.93	130.40	178.67	74.40	51.81	98.37
Alagoas	1,523	1,372	1,691	3,989	3,436	4,645	121.16	109.61	134.28	152.38	131.96	176.53	103.42	80.74	133.05
Amapá	94	82	107	561	440	711	84.91	74.68	96.05	132.05	104.78	165.70	71.85	44.71	102.72
Amazonas	1,242	1,093	1,399	4,604	3,826	5,491	153.02	135.57	170.76	192.88	161.41	227.96	65.34	43.30	89.35
Bahia	9,421	8,416	10,373	26,795	22,667	31,288	154.00	138.44	169.39	202.43	172.00	235.81	75.69	54.78	100.50
Ceará	4,066	3,563	4,542	14,504	12,350	16,965	113.75	100.48	127.02	190.16	161.77	222.32	130.13	96.36	170.24
Distrito Federal	1,534	1,406	1,664	5,495	4,700	6,402	211.47	195.45	228.85	223.77	191.63	258.05	46.12	29.05	66.17
Espírito Santo	2,561	2,339	2,808	7,890	6,737	9,115	180.80	165.41	197.17	210.46	180.57	242.19	55.31	37.12	74.64
Goiás	3,871	3,536	4,224	13,012	11,279	14,999	192.69	176.65	209.54	222.93	193.58	255.86	53.95	36.90	72.63
Maranhão	3,567	3,066	4,208	7,881	6,389	9,709	147.51	126.61	172.56	160.43	130.81	197.26	62.52	36.21	92.14
Mato Grosso	1,358	1,200	1,523	5,284	4,372	6,234	159.24	141.26	176.71	200.08	166.78	234.48	64.73	44.61	86.79
Mato Grosso do Sul	1,649	1,487	1,823	5,511	4,707	6,492	179.93	163.22	197.32	235.16	201.19	275.11	64.35	46.03	86.23
Minas Gerais	16,708	15,283	18,175	49,463	42,615	57,442	184.15	168.81	200.17	230.30	199.10	266.36	74.99	55.68	96.45
Paraná	10,536	9,617	11,492	31,366	27,135	36,159	225.91	207.17	245.92	279.26	242.40	320.95	60.15	43.16	78.51
Paraíba	2,212	1,979	2,464	5,935	4,879	7,233	114.52	102.66	126.91	165.57	136.52	201.49	84.69	58.63	113.20
Pará	2,915	2,560	3,272	9,381	7,632	11,451	133.43	117.96	149.53	168.59	138.85	205.40	68.69	46.35	95.74
Pernambuco	5,799	5,221	6,485	14,211	12,135	16,750	137.29	123.98	153.18	174.00	149.27	203.94	87.80	64.96	116.68
Piaui	1,486	1,264	1,711	4,153	3,497	4,864	114.95	98.29	132.36	153.93	129.97	179.83	67.03	39.61	96.86
Rio de Janeiro	24,355	22,636	26,372	54,467	48,241	61,620	268.25	250.73	288.40	295.87	262.52	334.55	63.00	46.88	79.50
Rio Grande do Norte	1,718	1,541	1,905	5,279	4,507	6,120	124.71	112.66	137.50	173.53	148.82	201.13	93.56	67.99	122.53
Rio Grande do Sul	18,016	16,440	19,832	39,670	33,364	46,942	297.06	271.35	324.56	303.18	256.00	357.04	32.15	16.30	49.89
Rondônia	574	510	639	1,869	1,571	2,159	135.42	121.43	149.79	144.77	122.59	166.71	58.30	39.99	77.04
Roraima	71	64	78	357	304	415	101.91	93.31	110.88	125.14	107.52	144.37	70.06	48.80	91.92
Santa Catarina	5,101	4,628	5,584	15,413	12,972	18,273	215.17	196.67	235.38	232.33	197.31	273.63	46.86	29.29	65.81
Sergipe	1,053	942	1,167	3,176	2,684	3,755	134.55	120.88	148.80	177.88	151.25	209.06	78.26	55.57	105.19
São Paulo	49,738	46,323	53,550	134,582	115,970	153,345	257.61	239.75	276.29	294.93	255.18	334.60	65.22	47.37	85.69
Tocantins	455	375	553	1,823	1,480	2,187	108.71	90.18	131.57	162.00	132.08	192.32	85.77	49.81	127.86

*Age-standardized rate; U.I.: uncertainty interval.
