# Supplementary material for: Mortality and years of life lost by colorectal cancer attributable to physical inactivity in Brazil (1990–2015): Findings from the Global Burden of Disease Study
Source: PLoS One. 2018 Feb 1;13(2):e0190943. doi: 10.1371/journal.pone.0190943 (PMC5794056; doi:10.1371/journal.pone.0190943)
Supplement: S3 Table — *Age-standardized rate; U.I.: uncertainty interval. (PDF) [file pone.0190943.s004.pdf]

Supplementary File 3.

Number and age-standardized rate (per 100,000 inhabitants) of deaths from physical inactivity due to all causes globally, in Brazil, and in the Brazilian states.

Mortality by physical inactivity due to all causes														
	1990			2015			1990			2015			Change (1990-2015)	
	Deaths	95% U.I.		Deaths	95% U.I.		Rate*	95% U.I.		Rate*	95% U.I.		%*	95% U.I.	
Global	1,031,823	790,04	1,271,248	1,605,494	1,265,202	1,955,709	19.46	14.90	23.97	21.78	17.16	26.53	33.64	30.35	38.00
Brazil	33,227	27,437	39,130	59,197	50,445	68,227	22.07	18.23	25.99	28.48	24.27	32.83	19.60	15.00	25.63
Acre	58	47	69	134	108	162	12.46	10.13	14.88	16.38	13.25	19.88	48.31	35.83	63.10
Alagoas	547	454	634	1,016	833	1,205	21.40	17.76	24.77	30.08	24.66	35.67	81.04	67.29	98.09
Amapá	30	25	37	99	75	127	9.52	7.77	11.48	12.63	9.60	16.21	26.89	15.48	39.92
Amazonas	241	201	288	573	471	694	11.09	9.26	13.30	14.34	11.78	17.38	41.07	30.40	53.77
Bahia	2,383	1,947	2,832	4,725	3,870	5,671	19.51	15.94	23.19	30.74	25.18	36.89	43.13	33.31	54.46
Ceará	1,152	945	1,38	2,677	2,185	3,275	17.71	14.53	21.21	29.70	24.23	36.33	65.60	53.25	80.43
Distrito Federal	182	147	216	492	413	588	10.58	8.56	12.55	16.56	13.88	19.78	22.71	15.44	32.75
Espírito Santo	501	405	598	1,04	835	1,253	18.75	15.13	22.35	26.04	20.92	31.37	17.12	9.59	26.55
Goiás	648	532	763	1,599	1,305	1,922	16.07	13.19	18.93	23.73	19.37	28.52	28.42	21.78	36.75
Maranhão	1,094	890	1,342	2,084	1,650	2,623	21.16	17.22	25.94	30.05	23.80	37.82	71.72	57.92	89.38
Mato Grosso	305	246	370	776	626	950	14.90	12.01	18.06	23.36	18.85	28.59	30.30	21.64	41.65
Mato Grosso do Sul	306	244	372	669	537	814	16.66	13.27	20.24	24.81	19.92	30.20	16.36	10.28	24.30
Minas Gerais	3,495	2,831	4,169	5,794	4,730	6,917	22.04	17.85	26.28	27.34	22.32	32.63	5.59	-0.09	12.91
Paraná	1,818	1,494	2,183	3,483	2,854	4,202	20.95	17.21	25.15	30.62	25.09	36.94	11.42	5.03	20.01
Paraíba	803	666	955	1,588	1,278	1,962	25.01	20.75	29.74	39.60	31.86	48.92	41.82	32.93	51.92
Pará	720	583	869	1,630	1,301	2,000	13.63	11.03	16.43	19.76	15.77	24.24	56.65	43.89	71.27
Pernambuco	1,736	1,422	2,061	3,027	2,455	3,653	23.47	19.22	27.86	32.01	25.97	38.63	48.33	37.92	60.12
Piaui	428	351	509	976	793	1,172	16.42	13.45	19.54	30.01	24.37	36.04	63.67	51.86	77.24
Rio de Janeiro	4,600	3,785	5,461	6,485	5,401	7,758	35.01	28.81	41.57	38.32	31.91	45.84	3.09	-2.63	9.80
Rio Grande do Norte	491	409	580	1,038	847	1,24	20.03	16.67	23.67	29.82	24.36	35.64	46.68	35.32	60.93
Rio Grande do Sul	2,573	2,049	3,077	3,935	3,183	4,732	27.44	21.86	32.81	34.15	27.63	41.07	-5.15	-10.36	2.18
Rondônia	151	123	180	378	307	453	13.69	11.14	16.33	21.05	17.05	25.18	58.99	48.41	70.88
Roraima	20	17	24	66	55	79	10.31	8.60	12.15	12.72	10.57	15.20	55.51	41.74	71.29
Santa Catarina	885	714	1,070	1,748	1,400	2,147	19.41	15.65	23.46	25.39	20.34	31.19	10.77	4.15	18.28
Sergipe	288	238	337	574	473	693	18.55	15.34	21.74	25.23	20.76	30.44	46.02	35.31	58.05
São Paulo	7,636	6,203	9,050	12,209	9,880	14,730	23.52	19.11	27.88	26.92	21.79	32.48	4.30	-0.71	10.49
Tocantins	130	102	164	380	302	468	14.26	11.21	17.97	24.73	19.68	30.45	59.14	43.89	77.35

*Age-standardized rate; U.I.: uncertainty interval
