# Supplementary material for: Mortality and years of life lost by colorectal cancer attributable to physical inactivity in Brazil (1990–2015): Findings from the Global Burden of Disease Study
Source: PLoS One. 2018 Feb 1;13(2):e0190943. doi: 10.1371/journal.pone.0190943 (PMC5794056; doi:10.1371/journal.pone.0190943)
Supplement: S4 Table — *Age-standardized rate; U.I.: uncertainty interval. (PDF) [file pone.0190943.s005.pdf]

Supplementary File 4.

Number and age-standardized rate (per 100,000 inhabitants) of DALYs from physical inactivity due to all causes globally, in Brazil, and in the Brazilian states.

DALYs by physical inactivity due to all causes															
	**1990**			**2015**			**1990**			**2015**			**Change (1990-2015)**		
	**DALYs**	**95% U.I.**		**DALYs**	**95% U.I.**		**Rate***	**95% U.I.**		**Rate***	**95% U.I.**		**%***	**95% U.I.**	
Global	22,318,505	17,061,985	27,529,902	34,603,468	26,904,922	42,282,104	620.98	476.83	764.34	514.91	402.35	628.90	17.90	13.84	22.87
Brazil	863,721	713,612	1,018,121	1,401,966	1,184,425	1,640,799	1,071.24	891.75	1,259.05	739.44	627.56	864.31	-2.73	-6.91	2.43
Acre	1,574	1,294	1,880	3,604	2,973	4,412	935.08	774.62	1,111.72	773.48	639.21	939.53	8.52	-0.85	18.50
Alagoas	13,836	11,490	16,071	25,543	21,008	30,844	1,169.64	980.23	1,354.66	1,016.15	839.68	1,220.03	40.50	29.22	54.14
Amapá	801	658	963	2,858	2,245	3,628	785.52	648.21	940.47	715.10	566.80	894.30	0.62	-9.29	11.25
Amazonas	6,592	5,559	7,798	15,888	13,050	19,399	919.32	779.14	1,082.11	723.44	599.67	874.73	3.17	-5.48	12.51
Bahia	59,482	48,721	70,973	110,758	91,579	132,644	999.63	822.59	1,189.55	856.43	711.57	1,022.42	14.52	5.50	25.24
Ceará	27,128	22,310	32,312	60,549	49,708	73,418	803.99	666.83	957.60	815.36	672.07	987.26	39.59	26.90	53.04
Distrito Federal	5,662	4,564	6,714	12,173	10,171	14,664	845.01	693.98	992.25	502.66	421.73	603.64	-17.92	-24.06	-10.74
Espírito Santo	13,633	11,247	16,326	25,899	20,884	31,277	1,018.87	838.25	1,211.81	702.82	568.44	845.90	-8.01	-15.14	-0.10
Goiás	18,463	15,058	21,909	42,022	34,498	50,376	973.56	800.32	1,147.78	737.52	608.12	880.31	0.76	-5.70	8.30
Maranhão	28,187	22,792	34,787	49,469	38,721	62,375	1,234.53	1,011.65	1,501.90	1,053.39	830.38	1,319.89	27.49	14.81	42.37
Mato Grosso	8,917	7,295	10,836	21,010	16,950	25,415	1,128.95	924.29	1,360.71	822.96	668.42	989.38	-4.47	-12.37	4.27
Mato Grosso do Sul	8,988	7,233	10,864	17,833	14,387	21,771	1,037.73	847.24	1,239.95	772.25	627.22	936.91	-6.46	-13.12	0.92
Minas Gerais	91,882	75,038	110,176	137,611	112,280	164,896	1,042.30	855.36	1,242.28	649.70	530.86	776.23	-12.84	-19.28	-5.66
Paraná	47,577	38,662	57,269	82,143	67,792	99,103	1,084.36	885.84	1,298.21	745.73	618.36	897.50	-10.94	-17.50	-3.40
Paraíba	18,523	15,279	21,790	36,273	28,933	45,107	1,000.03	829.20	1,176.28	1,032.22	828.40	1,278.79	31.83	20.78	43.73
Pará	18,826	15,251	22,665	42,363	33,350	52,222	940.77	767.05	1,126.54	811.33	647.60	992.60	15.11	4.55	26.96
Pernambuco	43,172	35,594	51,578	73,611	59,475	88,125	1,082.64	895.62	1,283.68	922.93	748.71	1,103.72	26.32	15.60	38.75
Piaui	10,843	8,852	12,849	23,733	19,309	28,248	886.79	727.97	1,046.30	906.68	740.88	1,076.87	27.55	16.75	39.37
Rio de Janeiro	124,351	102,783	147,449	154,233	128,881	182,716	1,380.31	1,143.67	1,629.59	836.69	699.84	989.81	-10.47	-16.81	-3.64
Rio Grande do Norte	11,161	9,244	13,166	23,694	19,187	28,429	847.00	703.70	996.12	800.42	651.30	957.71	31.37	20.30	43.99
Rio Grande do Sul	64,468	51,636	77,882	86,304	69,548	103,719	1,082.75	874.87	1,296.05	663.86	535.79	797.07	-20.65	-27.22	-13.23
Rondônia	4,637	3,786	5,486	10,607	8,605	12,792	1,225.38	1,009.72	1,440.06	856.84	700.60	1,024.90	3.51	-4.11	11.82
Roraima	627	521	746	2,030	1,679	2,402	995.76	834.93	1,167.73	747.53	624.17	878.49	3.92	-5.68	14.19
Santa Catarina	22,648	18,194	27,132	41,519	33,559	51,160	1,010.66	821.06	1,207.49	638.70	519.62	783.36	-14.07	-21.07	-6.55
Sergipe	6,841	5,715	8,074	14,143	11,691	17,083	937.85	784.61	1,106.61	837.59	697.05	1,001.83	20.41	10.89	30.70
São Paulo	201,468	163,549	240,703	276,566	224,067	332,834	1,073.55	877.93	1,271.66	610.34	496.25	733.46	-18.01	-24.10	-12.11
Tocantins	3,434	2,690	4,267	9,531	7,524	11,808	879.71	696.47	1,087.34	876.35	697.86	1,074.94	24.14	11.41	39.53

*Age-standardized rate; U.I.: uncertainty interval
